# Mild burn amplifies the locomotive depression in demyelinated mice without muscle pathophysiological changes

**DOI:** 10.1371/journal.pone.0308908

**Published:** 2024-10-07

**Authors:** Juquan Song, Amina EI Ayadi, Victoria G. Rontoyanni, Steven E. Wolf

**Affiliations:** Department of Surgery, the University of Texas Medical Branch at Galveston, Galveston, Texas, United States of America; Central University of Rajasthan, INDIA

## Abstract

**Introduction:**

Patients with mild burns take most accounts, however, the impact of mild burns is less known. Nerve destruction leads to muscle atrophy. We posit that even mild burn injury could worsen demyelinated nerves related to muscle pathophysiological impairment.

**Methods:**

Young adult C57BL/6 (male, n = 60) mice were randomly fed with either a 0.2% cuprizone diet or a regular rodent diet for 4 weeks. At week 5, all mice were then grouped into mild scald burn with 10% TBSA and sham injury groups. Mice received animal behavior tests and in situ muscle isometric force measurement before euthanasia for tissue collection.

**Results:**

Total horizontal ambulation and vertical activity were significantly reduced in mice with mild burn injury (p<0.05). Mice with the cuprizone diet had significantly less time to fall than those with the regular diet on day 7 after burn (p<0.05). No significant difference was found in gastrocnemius tissue weight among the groups, nor muscle isometric tensions (all p>0.05). The cuprizone diet increased the maximal phosphorylating respiration in mice muscle mitochondria (p<0.05). The muscle protein expressions of caspase 3, Fbx-32, and Murf1 significantly increased in mice with the cuprizone diet 3 days after burn (p<0.05). The signal expression of S100B significantly increased in mice with the cuprizone diet, and its expression was even greater on day 7 after burn injury. (p<0.05)

**Conclusion:**

The cuprizone diet-induced locomotion and cognitive disorders were amplified by the mild burn injury in mice, which is associated with muscle intracellular signal alterations. However, mild burn injury does not cause mouse muscle weight loss and function impairment. The potential risk of pre-existed neural impairment could be aware when patients encounter even small or mild burns.

## Introduction

Burn is one of the most common causes leading to patient injury, which could affect multiple organ systems. Especially, severe burns greater than 30% of total body surface area (TBSA) and over II-degree depth caused systemic responses and multiple system organ damages [1]. As the body barrier was destroyed, burn injury, especially severe burns could compromise neural [2, 3] and skeletomuscular systems [4]. The mechanism of severe burns impaired skeletal muscle with pathophysiological changes was mostly correlated to hyperinflammation and hypermetabolic status [5, 6]. With activated immune cell infiltration, released inflammatory media including cytokines disrupt muscle cell homeostasis, leading to a negative balance of protein degradation and cell death [5–7]. Besides the conventional mechanisms of hyper-inflammation caused muscle hyper-metabolic status, severe burns induced nerve systemic damage [8, 9], which also contributes to muscle wasting. Boston Burn Center showed in vivo that motor neuron damage from the spine participated in post-burn muscle wasting [10]. Another evidence includes that Irisn gene delivery improves muscle recovery in severely burned rats via alleviating peripheral neural damage [11]. As the neural system regulates skeletal muscle activity, peripheral nerve demyelination was found to induce muscle pathophysiological changes [12]. Mechanism of systemic inflammation driving neural degeneration was implied to the connection of demyelinated motor disorder, which contributed to muscle dysfunction and even muscle atrophy [13]. Severe burn caused peripheral neuropathy was notified [3]. It is true that neural damage leads to muscle function disorder and muscle atrophy [13, 14]. However, no such information was provided for a clear mechanism picture of those 3 elements’ relationship among burn, neural damage, and muscle pathophysiological changes. For instance, it is not clear whether the neural damage contributes to post-burn muscle dysfunction and mass loss.

The majority of patients are minor or mild burns. Patient epidemiological investigation in burn patients in South China showed that the average TBSA is 13.64%±16.83 with only 4.9% greater than 50% TBSA [15]. In our previous study of the recent 20 years’ burn patient population in the United States, we also found the majority of the patients (~72%) had minor size burned with 0–9% TBSA, and 13% of patients with 10–19% TBSA and rest 14% burned from 20–100% TBSA [16]. Clinical considerations of burn size and depth are applied for burn severity stratification, and most metabolic studies target severe burns with the small animal models of 20–30% TBSA of mice/ 40–60% of rats [17]. The II-degree burn from 10% to 20% TBSA could initiate a moderate systemic response which also requires fluid management [18]. However, discussions are still occurring about which moderate burn causes systemic response especially contributing to muscle pathophysiological changes. Pre-existing medical conditions often exist in burn patients, with mostly neurological disorders (40.2%), hypertension (16.6%), and diabetes (13.7%), which are associated with unfavorable patient progress [19]. We posit that pre-existed peripheral neural damage with demyelination caused muscle myofiber size decrease and function reduction, and these effects are worse when further encountering minor injury insult. Using a mild burn animal model, we investigated how demyelinated mice maintained muscle mass and function with the intracellular mechanism of myofiber homeostasis was disrupted following a scald burn with 10% TBSA.

## Methods

### Animal experiment ethics

All procedures followed the National Institutes of Health (NIH) and Animal Research: Reporting of In Vivo Experiments (ARRIVE) guidelines. The animal experiment protocols were approved by the Institutional Animal Care and Use Committee (IACUC) at the University of Texas Medical Branch at Galveston (UTMB) (APN#1811084A). 60 C57BL6 male mice (5 weeks old) were obtained from Charles River Laboratory (Wilmington, MA). Animals were housed in a temperature-controlled room with a 12-h light/ dark cycle with free access to laboratory diet and water with one-week acclimation.

### Experimental demyelinated mice

Animals were randomly divided into 2 groups on different diets and group housing during the 4-week-feeding period (3–4 mice per cage with ear-punched labels) for monitoring body weight and diet consumption. In Group 1 (n = 34), mice were given a 0.2% bodyweight cuprizone diet (#TD.140803, Envigo, Indianapolis, IN) in their chow for 4 weeks for demyelination development [20]. In Group 2 (n = 26), mice were given TEKLAD Global 18% Protein Diet (#T.2018.15, Envigo, Indianapolis, IN) as the diet comparable group. By recording the daily food consumption, the mouse consumed 0.72g/day cuprizone diet more than the regular diet. (p<0.05) At Week 5, all mice were further grouped into burn or sham injured group after excluding 5 (2 from the regular diet and 3 from the cuprizone diet) with body weight loss outliners (2 with malocclusions, 3 by unknown). The mice were housed individually after the burn procedure and continued the same type of diet till the end of the experiment. The whole experiment was conducted in two cohorts, from December 2019 to August 2020.

### Mouse mild burn procedure

Briefly, 37 mice were weighed and received a subcutaneous injection of 0.05 mg/kg buprenorphine 30 min ahead of the procedure. At the procedure started, animals were anesthetized with 2% isoflurane inhalation and shaved on the back, and 1 mL of 0.9% saline injection was subcutaneously administered around the spine. Mice were then placed in a mold with an opening exposing about 12.5% body surface, in a supine position. Mice were then immersed in 98°C water for 10 seconds on the dorsal side to create a full-thickness scald burn. Right after the injury, the animals received 1 mL of intraperitoneal lactated Ringer’s solution for fluid resuscitation. All animals survived after the burn procedure. Planimetry measurement of mouse burn size was performed with plastic wrap marked after the burn procedure and re-estimated at day 3 post-burn [21]. The average percentage of burn size was 10.7±1.3% TBSA (n = 17). 18 sham animals received the same steps of the analgesics and anesthetics but did not receive a scald burn or resuscitation.

*Animal behavior test*s were performed at the Rodent *In vivo* Assessment (RIVA) core facility at UTMB.

1. *Open Field Test*: On day 6 after burn, mouse locomotor activity was monitored under low light conditions using a modified open field system (San Diego Instruments, CA). Mouse was transported into individual Clear Plexiglas chambers (40×40×40 cm) during 60 minutes’ stay. The chambers were surrounded by a 4×4 photo beam matrix positioned 4 cm from the chamber floor. A second row of photo beams was positioned approximately 16 cm above the chamber floor for vertical activity assessment. The control software counted total horizontal activity as well as vertical beam interruptions and stored the counts in 5-min bins; these data are reported as total ambulation [22, 23]
2. *Rotarod Test*: Animals were trained on the rotarod treadmill (MK-670, Muromachi Kikai, Japan) three times before burn or sham. After the acclimation training on the stationary rod, the animals were tested for balance at a constant speed of 4 RPM on day 6 after the burn injury, and the times to fall from the stationary rod were recorded four consecutive times [24].

### Muscle isometric force measurement

In situ gastrocnemius isometric force measurement was followed previous study [25]. Isometric contractile properties of the gastrocnemius muscles were measured on days 3 and 7 after burn (ASI Dynamic Muscle Control v5.300, Aurora Scientific, Canada). Under general anesthesia, the mouse was laid in a prone position on the warm platform with 37°C water circulated. The gastrocnemius muscle was gently dissected free of surrounding musculature, skin, and fascia to maintain the neurovascular pedicle as well as the proximal and distal attachments. The Achilles tendon was sutured and attached to the lever arm of a dual-mode servo muscle lever system (mod 305c, Aurora Scientific, Canada). The femur was secured to the platform. Electrodes were implanted into the distal end of the severed sciatic nerve. A single 200 Hz twitch was stimulated with an impulse duration of 0.2 ms at 10 mA. The muscle was then stretched 0.2 mm and stimulated again with a 25-second break between stimulations. This pattern was continued until less than a 2% change between twitches was detected, indicating the optimal length (Lo) of the muscle. The maximum twitch (Pt), force frequency, tetanic (Po), and fatigue parameters were then consequently measured at Lo. Isometric tetanic function was stimulated at 20, 40, 80, 100, 150, 180, and 200 Hz with impulse duration of 0.2 ms, 75 pulses per train, at 10mA, for a total of 1 second. Po was measured three times with an off-tension recovery period of 2 minutes between stimulations. Relaxed muscle fiber at the baseline and applied mineral oil during the relaxation time.

*Skeletal muscle mitochondrial respiratory capacity* was measured via high-resolution respirometry as previously described [26]. In brief, about 20mg of fresh gastrocnemius tissue was immersed in preservation buffer on ice before being transferred into the O2K chamber with the polygraphic oxygen sensors controlled by DatLab software (Oroboros Instruments, Innsbruck, Austria) and containing 2 mL respiration buffer. Preservation buffer includes 2.77 mM CaK2EGTA, 7.23 mM K2EGTA, 50mM MES hydrate, 20mM imidazole, 20mM taurine, 15mM Na2Phosphocreatine, 6.56 mM MgCl2·6H20, 5.77 mM Na2ATP, and 0.5 mM dithiothreitol; pH 7.1. Respiration buffer contains 0.5mM EGTA, 60mM lactobionic acid, 3 mM MgCl2·6H20, 20 mM taurine, 10mM KH2PO4, 20 mM HEPES, 110 mM sucrose, and 1g/L bovine serum albumin. All experiments were performed within a range of 200–450μM of oxygen concentration to ensure that oxygen consumption and diffusion would not be limited to respiration. Mitochondrial respiratory capacity was determined by the sequential addition of substrates and uncouplers. State 2 (leak) respiration supported primarily by electron flow through complex I of the respiratory chain (LI) was achieved by the titration of 1.5mM octanoyl-l-carnitine, 5mM pyruvate, 2mM malate, and 10mM glutamate into the O2K chamber. Electron transfer was then coupled to phosphorylation by the addition of 5mM ADP, inducing coupled state 3i respiration with electron transfer supported by Complex I (PI). 10mM succinate was added to the O2K chamber to induce maximal state 3i+ii respiration with parallel electron input from Complex I and II (PI+II). 10μM cytochrome C was added to assess the competence of the outer mitochondrial membrane. The absence of a significant increase in respiratory flux following the addition of cytochrome C indicates that the outer mitochondrial membranes are intact. Finally, oxidative phosphorylation was uncoupled by the titration of carbonyl cyanide m-chlorophenylhydrazone (CCCP) to a final concentration of 5μM to assess maximal electron transfer capacity (E). All chemicals were purchased from Sigma-Adrich (St. Louis, MO) unless specified.

*Western blot assay* was followed by the previous publication [27]. Briefly, 20 mg of frozen gastrocnemius muscle tissue was homogenized with 0.1mm Zirconium Homogenizer Beads (CPI international) for protein extraction. Tissue lysates were prepared using T-PER tissue protein extraction reagent (Thermal Scientific, Rockford, IL). Protein concentrations were examined by the protein assay kit (BioRad, Hercules, CA) based on the Bradford dye-binding method. Using the Bio-Rad Mini-Protean system (Bio-Rad, Hercules, CA), approximate 20μg of protein samples were applied on Sodium dodecyl-sulfate polyacrylamide gel electrophoresis (SDS-PAGE, 4–20%) and the separated protein components were transferred to a Polyvinylidene difluoride (PVDF) membrane. The membranes were then blocked by the nonspecific binding background with 5% bovine serum albumin (BSA) at room temperature for 1 hour and subsequently probed with primary antibodies at 4°C overnight. The membranes were then incubated with horse-radish peroxidase (HRP)-conjugated secondary antibodies for 1 hour. After rinsing off the secondary antibodies, the blots were applied for protein signal detection using SuperSignal West Pico PLUS Chemiluminescent Substrate (Thermo Scientific, Rockford, IL). The stained blots were examined using the ChemiDoc™ Touch Imaging System (BioRad, Hercules, CA). All of antibodies were from Cell Signaling Technology (CS#) (Danvers, MA) or Abcam Biotechnology (#ab)(Cambridge, United Kingdom), including Mruf1/2/3 (#ab172479), Fbx32 (#ab74023), caspase 3 (CS#9662), PCNA (CS#13110), MBP (#ab40390), and S100B (#ab52642). To analyze the blots, BioRad Image lab software was used to quantify band intensity and calculate the ratio of the target protein compared to the loading control GAPDH (CS#5174).

### Statistical analysis

Data were captured in Excel and analyzed by using GraphPad Prism v.9.4.0 (San Diego, CA) and SigmaPlot 14.0 (Systat Software. Inc). Two-way ANOVA with post-hoc analysis was applied. Significance comparing any two groups was calculated by Students’ unpaired t-test with or without Welch’s correction or Mann-Whitney U test. Data were presented as mean values ± standard error mean (SEM). Significance was accepted at p-value <0.05.

## Results

### Demyelinated mice’s body weight changes during the experiment

**Fig 1:**
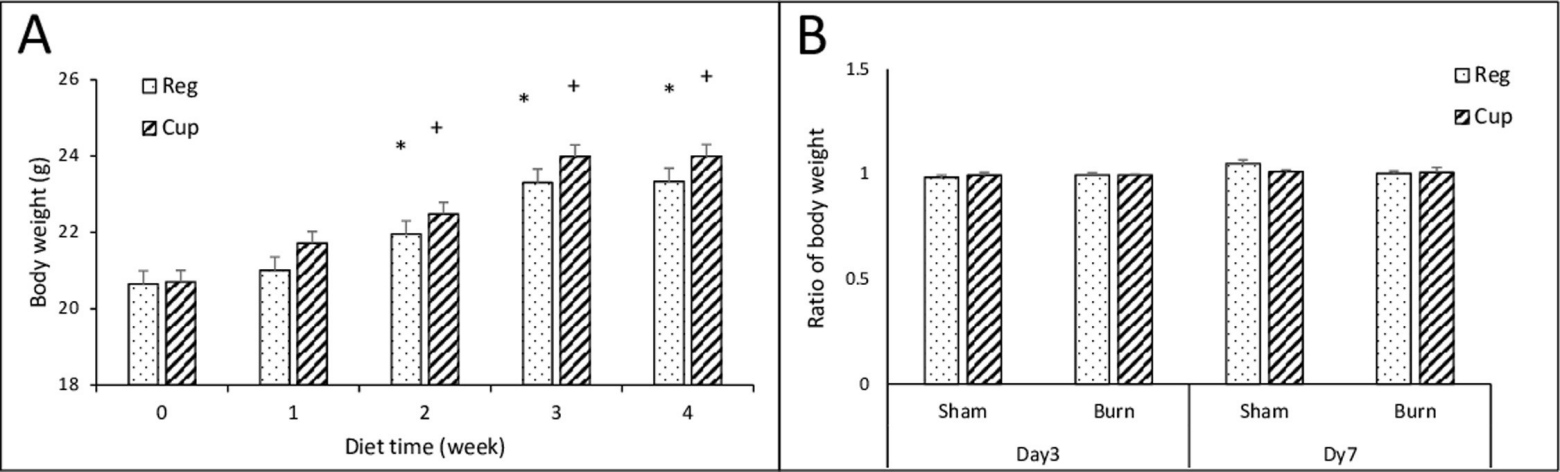
Animal body weights during the experiment. A) mouse body weight significantly altered over 4 weeks of feeding between 18% protein regular diet (Reg, n = 24, dot bar) and 0.2% cuprizone diet (Cup, n = 31, slant bar) (p = 0.012). *, p<0.05, Reg diet vs. week 0; +, p<0.05, Cup diet vs. week 0. Two-way ANOVA; B) Mouse body weight changes (normalized to individual body weight before sham and burn procedure) with regular diet (Reg, dot bar) and cuprizone diet (Cup, slant bar) in the sham and mild burn groups at 3 and 7 days after injury.

Compared to each body weight at the beginning of feeding, the mouse had significant body weight in both groups. Mice fed with the regular diet had gained greater body mass in 3^rd^ week with a difference of the mean of 2.663 (vs. week 0, p<0.001); mice with the cuprizone diet had greater body mass a week earlier with a difference of mean of 1.784 (week 2 vs. week 0, p<0.001). Mice with the cuprizone diet gain more weight during the 4 weeks. (Cup. vs. Reg. diets, p<0.05) [**Fig 1A**].

After 4 weeks feeding, all mice received either a burn or sham procedure. Examining the ratios of mouse body weight normalized to body weight at day 0 of injury, we did not find mouse body weight changes altered significantly with either cuprizone diet or regular food in both sham and burn-treated groups [**Fig 1B**].

### Animal behavior altered with the cuprizone diet, especially in response to burn injury

**Fig 2:**
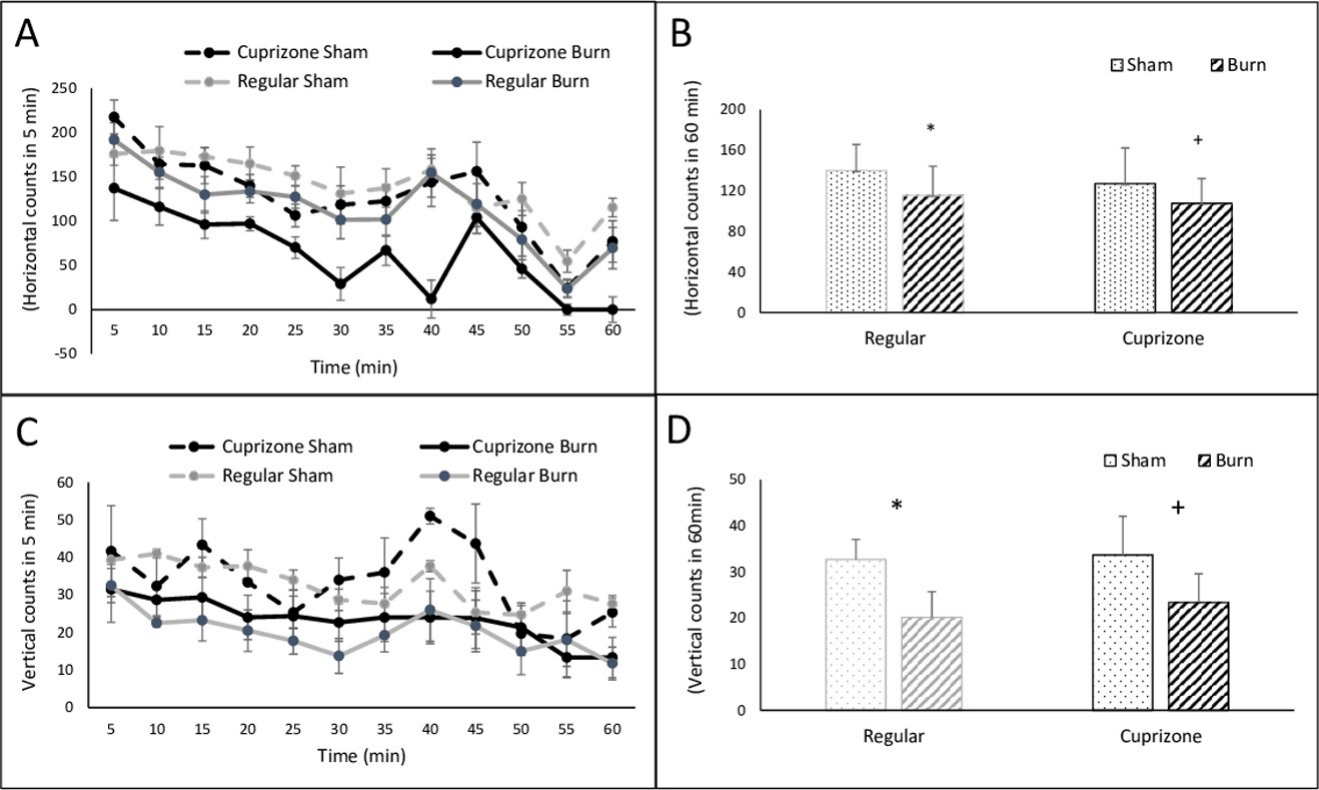
Animal open field test including A) the 5-minute alteration of horizontal ambulation over 60 minutes of testing; B) total horizontal ambulation over 60 minutes. C) the 5-minute alteration of vertical ambulation over 60 minutes of testing; D) total vertical ambulation over 60 minutes of testing. Reg diet: Sham, n = 3, and burn, n = 4; Cup diet: sham, n = 3 and burn, n = 6; *, p<0.05, sham vs. burn within Reg diet; +, p<0.05, sham vs. burn within Cup diet. Two-way ANOVA.

The open field was to test anxiety-like behavior as well as locomotor activity [28]. Analyzing total horizontal activity, the results from 5-minute intervals of consecutive beam counts clearly showed that injured mice with the cuprizone diet decreased the most with about 30% activity at the first 40 minutes of measurement [**Fig 2A**]. The 5-minute interval of consecutive beam counts of vertical activity also disclosed that mice with both diets had dropped activities with mild burn insult within the 60-minute test [**Fig 2C**]. The average of beat counts showed that mice significantly decreased both horizontal and vertical activities after burn injury in both regular and cuprizone diets. (p<0.05) [**Fig 2B and 2D**] The study demonstrates that even mild burn at 6 days’ the mouse presented anxiety-like behavior.

**Fig 3:**
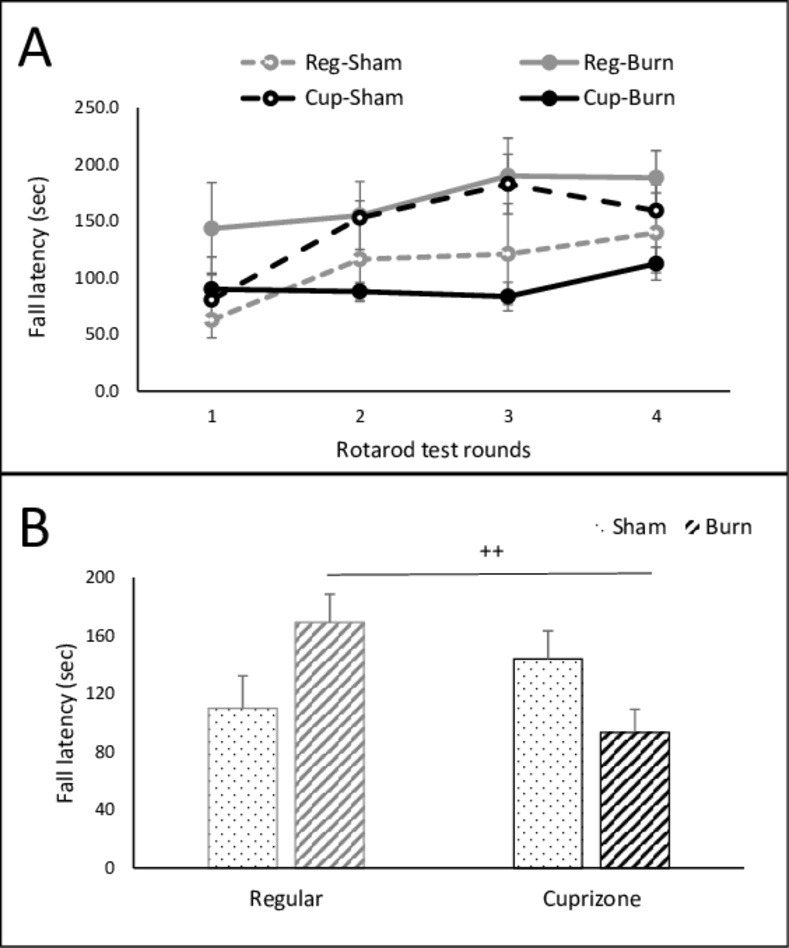
Animal behavior rotarod test A) 4 rounds of mice rotarod test of the latency to fall on the trials between sham and burn at day 7 after injury. B) the average of mouse fall latency in four groups. Reg.Sham, n = 3, and burn, n = 4; Cup diet: sham, n = 3 and burn, n = 6; ++, p<0.05, Reg diet vs. Cup diet in burned mice. Two-way ANOVA.

In the rotarod test, the latency to fall significantly increased in the 2^nd^ trial (153±15) when compared to the 1^st^ trial (80±24) in mice with the cuprizone diet. (p<0.05) There was no significant difference between sham groups with two diets (110±22s in regular, 144±19s in cuprizone). Within the 2^nd^ and 3^rd^ respective trials, significant alterations were observed in either burned mice between cuprizone and regular diets, or cuprizone-fed mice between sham and burn conditions [**Fig 3A**]. When the mouse was insulted by a mild burn, an injured mouse with the cuprizone diet significantly reduced the stay time (93±16s) when comparing to injured mouse with regular diet (169±19s, p<0.05). The latency to fall was not even improved in the injured mouse with the cuprizone diet during the repeated trials. The results disclosed a fragile cognitive status in mice with a cuprizone diet, and the defective locomotion response was further displayed following even a mild injury [**Fig 3B**].

### Mouse gastrocnemius muscle tissue weight and isometric force

No significant difference was observed in mouse gastrocnemius tissue weight between the two diet groups under sham and burn conditions. By measuring myofiber optimal length (Lo), tetanic force (Po), and twitch force (Pt), we did not find any significant alterations, either between two diet groups or between two burn and sham conditions. Muscle fatigue estimation including minimal tension (Min), maximum tension (Max) and fatigue index (FI) did not demonstrate any significant muscle strength loss in cuprizone diet mice following mild burn injury. All data are presented in **Table 1**.

**Table 1:** Mouse muscle mass and isometric tensions in response to diet and mild injury.

Diet	Reg	Reg	Cup	Cup	Reg	Reg	Cup	Cup
Injury	Sham	Burn	Sham	Burn	Sham	Burn	Sham	Burn
Time after burn (day)	3	3	3	3	7	7	7	7
n =	3	4	3	5	3	4	3	4
Tissue weight (mg)	120.13±3.39	132.73.13±8.67	127.88±7.77	122.33±4.12	116.80±8.80	126.41±4.41	133.77±2.56	115.33±2.14
Lo(mm)	17.10±1.83	17.65±0.96	17.30±0.60	18.66±0.47	16.50±0.07	16.06±1.24	16.86±0.18	15.95±0.77
Twitch tension(Pt) (g)	75.50±5.02	70.24±4.52	77.52±5.49	80.01±1.36	76.03±13.14	91.52±1.81	87.82±7.01	79.09±6.04
Tetanic tension (Po) (g)	317.58±29.21	380.40±38.83	382.72±25.98	389.13±45.52	345.96±36.53	400.62±57.20	444.24±14.13	370.02±19.41
Fatigue								
Max (g)	217.33±48.82	251.79±16.20	251.38±10.71	244.81±24.57	242.65±16.73	255.33±30.27	297.44±14.17	252.61±21.37
Min (g)	23.05±2.62	42.73±6.42	31.67±2.00	38.071±3.76	24.96±5.25	38.89±8.68	45.42±8.90	34.46±5.89
FI (%)	89.12±1.24	83.27±1.88	87.35±1.34	84.36±0.95	89.51±2.89	85.44±2.00	84.38±3.87	86.36±2.03

Lo- optimal length; FI–fatigue index, Two-way ANOVA

### Mitochondrial respiratory capacity in gastrocnemius muscles

We examined the effects of burn injury and cuprizone diet on mitochondrial respiratory states in gastrocnemius muscles at 3 and 7 days after burn using ANOVA models for all 2-way interactions. Leak (State 2i) respiration was lower at 3 days after burn fed with regular diet than cuprizone diet in mouse muscle tissues (14.5±5.0 vs. 22.3±4.8 pmol/sec/mg; p = 0.01), independent of burn injury, but did not significantly differ between groups at 7 days after burn (23.1±4.0 vs. 21.0±5.7 pmol/sec/mg). The cuprizone diet increased maximal phosphorylating respiration (State 3i+ii) compared to the regular diet at 3 days after burn (103.0±34.8 vs. 48.0±19.2 pmol/sec/mg; p = 0.02), with no differences between diets at 7 days after burn (76.1±29.0 vs. 74.3±20.8 pmol/sec/mg); and these effects were independent of burn injury. Similar were the responses for maximal uncoupled respiration (ETS capacity: cuprizone vs regular diet, 112.3±36.8 vs. 51.6±19.5 pmol/sec/mg at 3 days after burn; 79.0±26.7 vs. 78.5±16.8 pmol/sec/mg at 7 days after burn; p = 0.01), independent of burn injury as well. In summary, mild burn injury did not significantly impact any of the respiratory states or indices of coupling control in mouse gastrocnemius muscle.

### Muscle protein signal altered in response to diet and mild burn

**Fig 4:**
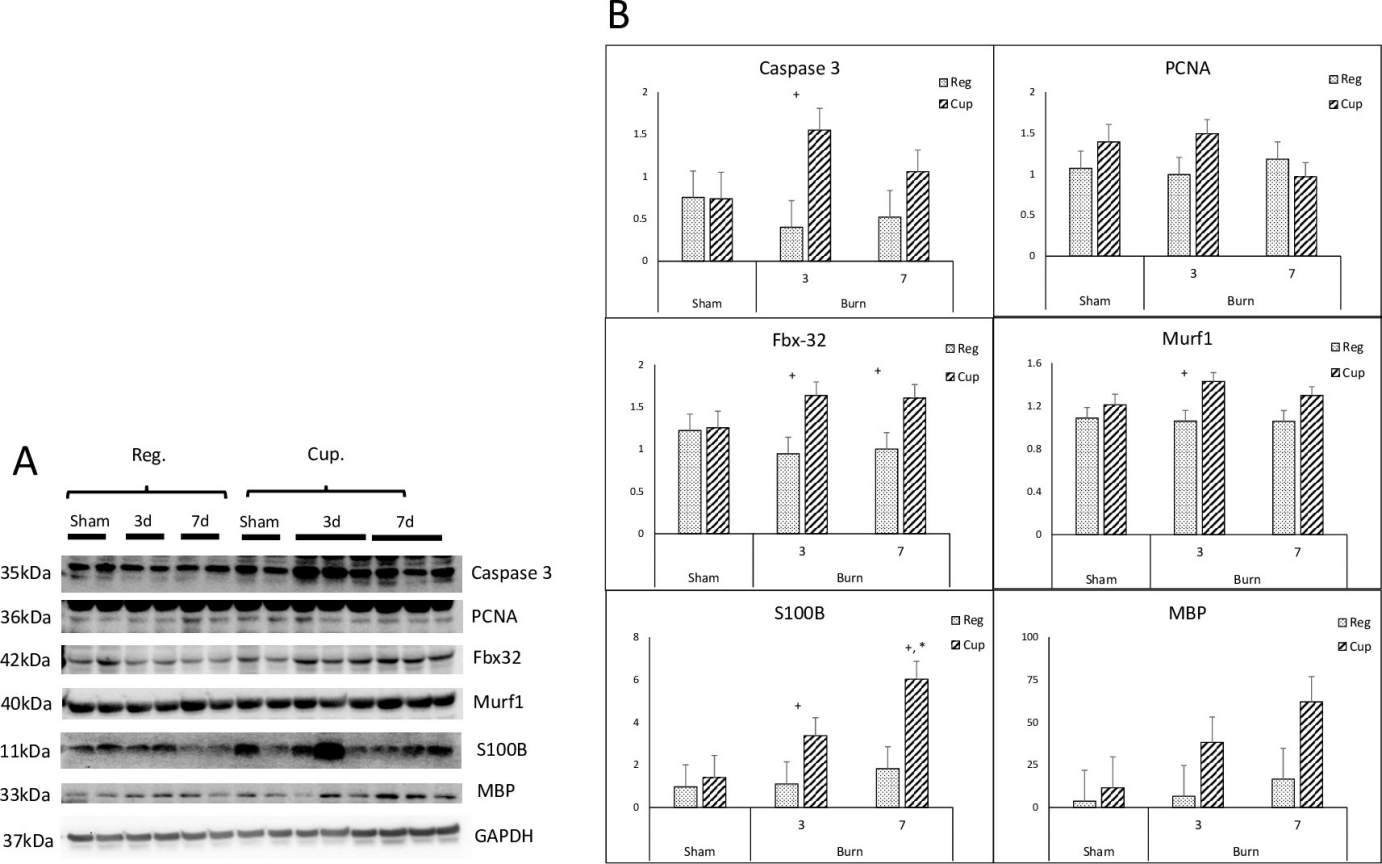
Mouse muscle protein signal regulation of muscle homeostasis A) western blot images of caspase 3, PCNA, Fbx32, Murf1, S100B, and MBP; GAPDH served as housekeeping protein; B) statistical plots of protein signals expressions of caspase 3, PCNA, Fbx32, Murf1, S100B, and MBP. Reg diet: Sham, n = 4, Burn 3days, n = 4; Burn 7 days, n = 4; Cup diet: Sham, n = 4, Burn 3days, n = 6; Burn 7 days, n = 6. +, p<0.05, Cup diet vs. Reg. diet; *, p<0.05, Burn injury vs. Sham.

Western blot data analysis showed that cellular apoptotic signal caspase3 significantly increased about 1.5 times in cuprizone diet rat at day 3 after injury (1.55± 0.26 in Cup. vs. 0.40±0.31 in Reg. diet; absorbance ratio, p = 0.031); muscle-specific E3 ubiquitin ligases Murf1 (1.43± 0.08 in Cup. vs. 1.06±0.10 in Reg. diet) and Fbx32 (1.64± 0.16 in Cup. vs. 0.94±0.19 in Reg. diet) significantly increased their expressions in cuprizone diet 3 days after burn injury (cuprizone vs. regular diet, p<0.05); cell proliferation marker PCNA expression has insignificant altered either respond to diet or burn injury. S100B and MBP are related to neural damage, and we found that S100B significantly increased its expression in cuprizone-dieted rats 3 days after burn (3.37± 0.84 in Cup vs. 1.11±1.03 in Reg diet, +, p = 0.008). At the later time of 7 days after injury, s100B expression significantly increased in rats with cuprizone diet when compared to those sham injured (6.02±0.84 in burns vs. 1.41±1.03 in sham, p = 0.034) **[Fig 4].** In summary, protein signal alterations were significantly responded to by the diet feeding which is independent of burn injury, suggesting the impact of a cuprizone diet on muscle tissue homeostasis regulatory signals.

## Discussion

In this study, we found that mice with a 4-week cuprizone diet gained heavier body weight than those with an 18% protein regular diet. There were no apparent animal abnormalities in both behavior and muscle homeostasis at the beginning of the burn procedure. However, the injury procedure of 10% TBSA mild burn impacted mouse horizontal and vertical ambulation, indicating animal cognitive depression and locomotor disorder related to mild burn. Interestingly, mice with a cuprizone diet had a significantly shorter time of stay on the rotarod after injury while mice with a regular diet did not. Further examining mouse skeletal muscle, we did not find any significant changes in muscle mass and function in response to diet and burn injury. However, we found that muscle intracellular signal and mitochondrial changes related to the cuprizone diet rather than a mild burn injury.

We were not surprised by the different responses of muscle homeostasis following mild burn in the current study after comparing it to previous observations in the scenery of a large-size severe burn injury. From our previous studies with full-thickness scald burn with greater burn size of 25% TBSA (including both ventral and dorsal sides of body area burned), injured mice lost 10–15% body weight, as well as a significant hind limb muscle mass loss at 3 days after severe burn [25, 27]. At the same time, the muscle homeostasis regulatory signals including caspase 3 and PCNA were significantly altered. In the current study, we did not find the significant losses of animal body weight, muscle tissue weight, and muscle function in mice following a mild burn of 10.7% TBSA, in both regular and cuprizone diets. Correspondingly, the protein signals did not change in mice with the regular diet either. Indeed, muscle homeostasis regulatory pathways were affected by the injury severity. We previously observed that activated signal expressions of caspase 3, Murf1, and PCNA, and myogenic regulator factors were associated with the injury severity of TBSA [29]. The current study again confirms the prominent impact of severe burn injury on muscle wasting, but not from mild burn with 10% TBSA.

In this study we found that muscle mitochondrial function was also altered in response to cuprizone diet, which seems in cooperated with muscle homeostasis signal changes. In other words, we found that muscle respiration capacity increased in cuprizone-fed mice following burn injury, along with the elevated signal protein expressions of caspase 3, Fbx-32, and Murf1. In regular diet-fed mice, we did not see those changes. The impact of the diet is independent of the mild burn. The muscle intracellular alterations are also correlated to our rotarod test observation, suggesting the effect of the cuprizone diet becomes prominent after encountering mild injury.

Burn injury with a deeper thickness or greater size, could lead to impaired peripheral nerves underneath wound sites locally and systemic neural damage. In our pilot study, we observed the morphological changes of sciatic nerve and muscle-increased MBP expressions after scald burns on mice. In this study, we did not examine the innervated nerve. Instead, we examined S100B and MBP protein expressions in mouse muscle by western blot analysis. S100B calcium-binding protein is applied as a parameter of glial activation and/or death in many disorders of the nervous system (CNS) [30], and is also correlated with demyelination in multiple sclerosis [31]. In this study, we chose S100B and another myelin basic protein (MBP) and found that both expressions increased in injured mice fed with a cuprizone diet but not in a regular diet after mice were injured. The results indicate that the demyelination destructive potential was exemplified even by mild burn injury. The significant elevation of S100B also indicated that the s100b is more sensitive even in muscle tissue, which might be served as the predictive potential in the future.

The study of behavior examination was originally designed to provide extra evidence of muscle pathophysiological response. As the skeletal muscle is the part of supporting the intact movement, the data showed clearly and objectively that cuprizone diet-induced central or peripheral neural demyelination damage, and we also captured the stress signs from burned animals. However, we did not find that demyelination impacts muscle pathophysiological changes within a week after injury. In future, we will extend our observational period so that we can evaluate the effect more completely.

The cuprizone diet animal models have been well described and established [32]. However, the tissue responses are different from animal species [33], feed periods [34], and feed formulation [35]. Withdrawn the drug, animals can be self-recovered from demylination. In this study, we found that 5-week-old male mice gained more body weight during the 4-week feeding period of a 0.2% cuprizone diet. However, there are no other behavior abnormalities or muscle pathophysiological changes observed in sham groups with different diets. The animal responses may be explained by the above reasons including our animal mice strain, age, and feeding status. However, the demyelination detrimental effect of the cuprizone diet can still be captured when mice encounter a mild burn injury.

As for the clinical relevance of the study, we should be aware of patients with those pre-existing diseases as their worse progress even with minor accident injuries. Our study showed that the cuprizone diet caused intracellular molecular changes in mouse muscle, however, the animal cognitive destruction and locomotor disorder became prominent when they received even mild burn injury. Clinically, it reminds that patients with previous unnoticeable demyelination or other neural impairment could be aware of when encountering even minor burn injury. In addition, as others reported, S100B might have a strong predictive potential and could be further investigated for progress.

In conclusion, the amplified effect of mild burn on demyelination impairments was observed in mousse muscle homeostasis and mitochondrial activity. The Demyelinated mice only presented changes in molecular levels when receiving mild burn insult, without changing pathophysiological within 7 days after injury. locomotive dysfunction was also amplified in demyelinated mice after burn injury, indicating a fragile responsive balance and a potential risk when suffering a minor injury.

## Supporting information

S1 Raw images Original western blots and images of mouse muscle protein signals presented in Fig 4. (PDF)

S1 Dataset Minimal datasets of the values processing for figures and tables. (XLSX)
