# Supplementary material for: Mild burn amplifies the locomotive depression in demyelinated mice without muscle pathophysiological changes
Source: PLoS One. 2024 Oct 7;19(10):e0308908. doi: 10.1371/journal.pone.0308908 (PMC11458009; doi:10.1371/journal.pone.0308908)
Supplement: S1 Raw images — (PDF) [file pone.0308908.s001.pdf]

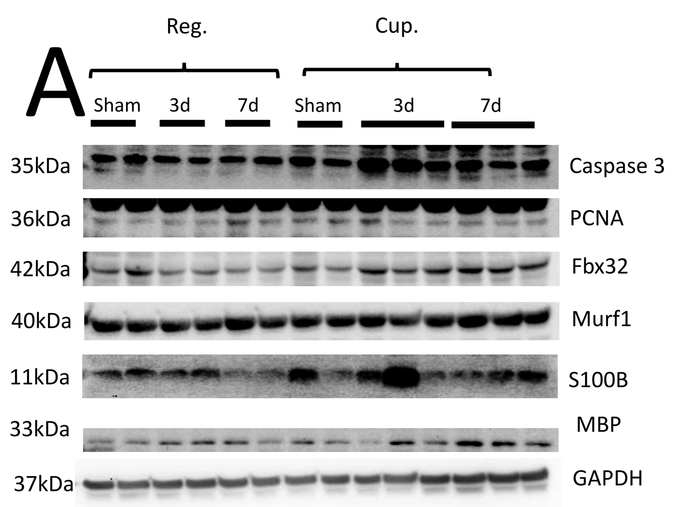

Figure 4A

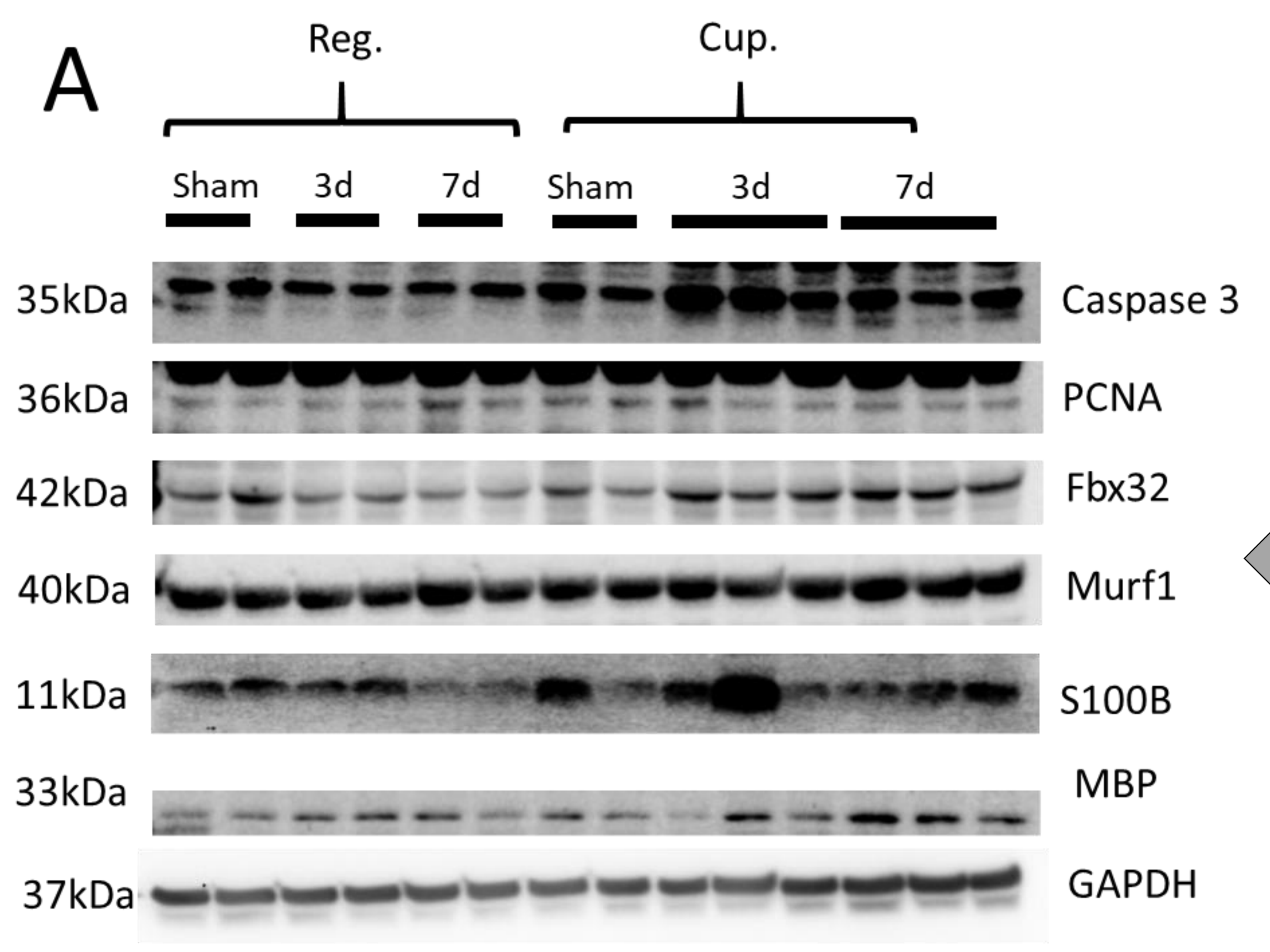

File name

SongJ 2020-02-26 11h47m07s-sv1-gstr-caspase3 gel 1, 2 1st round
SongJ 2020-02-28 11h35m46s-sv1-gastr-pcna
SongJ 2020-03-02 11h56m29s-sv1-fbx32
SongJ 2020-02-27 10h38m24s-sv1-gastr-murf1
SongJ 2020-03-06 11h41m15s-sv1-18%-S100b
SongJ 2020-03-09 11h10m11s-sv1-18%-mbp
SongJ 2020-03-02 11h49m41s-sv1-gapdh

Sample with exp. conditions, protein concentration, amount

				BCA	
SV1	Assgined group			(mg/ml)	20ug=ul
#03	excluded	XX	18% diet	-0.74898	
#04	sham	7day	18% diet	2.705859	7.39
#05	sham	7day	18% diet	4.791439	4.17
#09	sham	7day	18% diet	4.789326	4.18
#13	sham	7day	18% diet	3.98531	5.02
#01	Burn	3day	18% diet	5.467615	3.66
#07	Burn	3day	18% diet	4.45969	4.48
#08	Burn	3day	18% diet	3.498252	5.72
#11	Burn	3day	18% diet	4.205068	4.76
#02	Burn	7day	18% diet	4.18605	4.78
#06	Burn	7day	18% diet	3.678918	5.44
#10	Burn	7day	18% diet	4.971048	4.02
#12	Burn	7day	18% diet	4.518856	4.43
#17	sham	7day	0.2% cup	4.623452	4.33
#19	sham	7day	0.2% cup	5.220388	3.83
#25	sham	7day	0.2% cup	4.807287	4.16
#27	sham	7day	0.2% cup	3.339774	5.99
#30	sham	7day	0.2% cup	5.722238	3.50
#14	Burn	3day	0.2% cup	5.25948	3.80
#16	burn	3day	0.2% cup	5.019648	3.98
#20	Burn	3day	0.2% cup	3.920862	5.10
#23	Burn	3day	0.2% cup	5.434863	3.68
#24	Burn	3day	0.2% cup	3.391543	5.90
#28	Burn	3day	0.2% cup	3.327095	6.01
#15	Burn	7day	0.2% cup	4.847435	4.13
#18	Burn	7day	0.2% cup	5.111566	3.91
#21	Burn	7day	0.2% cup	4.279024	4.67
#22	Burn	7day	0.2% cup	2.923503	6.84
#26	Burn	7day	0.2% cup	4.658317	4.29
#29	Burn	7day	0.2% cup	4.391016	4.55

Sample loading info. For WB analysis

25-Feb		24-Feb	4-20% gel	15well											
2.5gel															
protien	gel 1,3	lane 2	lane 3	lane 4	lane 5	lane 6	lane 7	lane 8	lane 9	lane 10	lane 11	lane 12	lane 13	lane 14	lane 15
		#04	#05	#01	#07	#02	#06	#17	#19	#14	#16	#20	#15	#18	#21
		sham	sham	Burn	Burn	Burn	Burn	sham	sham	Burn	burn	Burn	Burn	Burn	Burn
				3day	3day	7day	7day	7day	7day	3day	3day	3day	7day	7day	7day
	gel 2, 4	lane 2	lane 3	lane 4	lane 5	lane 6	lane 7	lane 8	lane 9	lane 10	lane 11	lane 12	lane 13	lane 14	lane 15
		#09	#13	#08	#11	#10	#12	#25	#27	#23	#24	#28	#22	#26	#29
		sham	sham	Burn	Burn	Burn	Burn	sham	sham	Burn	Burn	Burn	Burn	Burn	Burn
		7day	7day	3day	3day	7day	7day	7day	7day	3day	3day	3day	7day	7day	7day
		3/5/2020	rerun	14ul	2x 18% gels		for	**mbp**	**s100b**						
	gelA	lane 2	lane 3	lane 4	lane 5	lane 6	lane 7	lane 8	lane 9	lane 10	lane 11	lane 12	lane 13	lane 14	lane 15
		#04	#05	#01	#07	#02	#06	#17	#19	#14	#16	#20	#15	#18	#21
		sham	sham	Burn	Burn	Burn	Burn	sham	sham	Burn	burn	Burn	Burn	Burn	Burn
				3day	3day	7day	7day	7day	7day	3day	3day	3day	7day	7day	7day
	Gel B	lane 2	lane 3	lane 4	lane 5	lane 6	lane 7	lane 8	lane 9	lane 10	lane 11	lane 12	lane 13	lane 14	lane 15
		#09	#13	#08	#11	#10	#12	#25	#27	#23	#24	#28	#22	#26	#29
		sham	sham	Burn	Burn	Burn	Burn	sham	sham	Burn	Burn	Burn	Burn	Burn	Burn
		7day	7day	3day	3day	7day	7day	7day	7day	3day	3day	3day	7day	7day	7day

Fig 4A

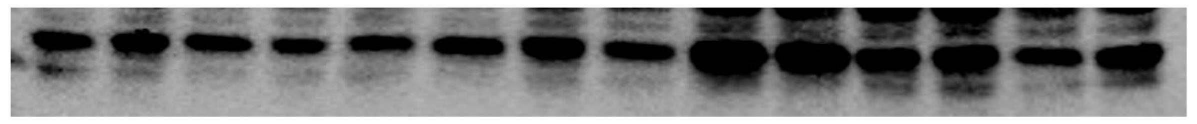

Caspase 3

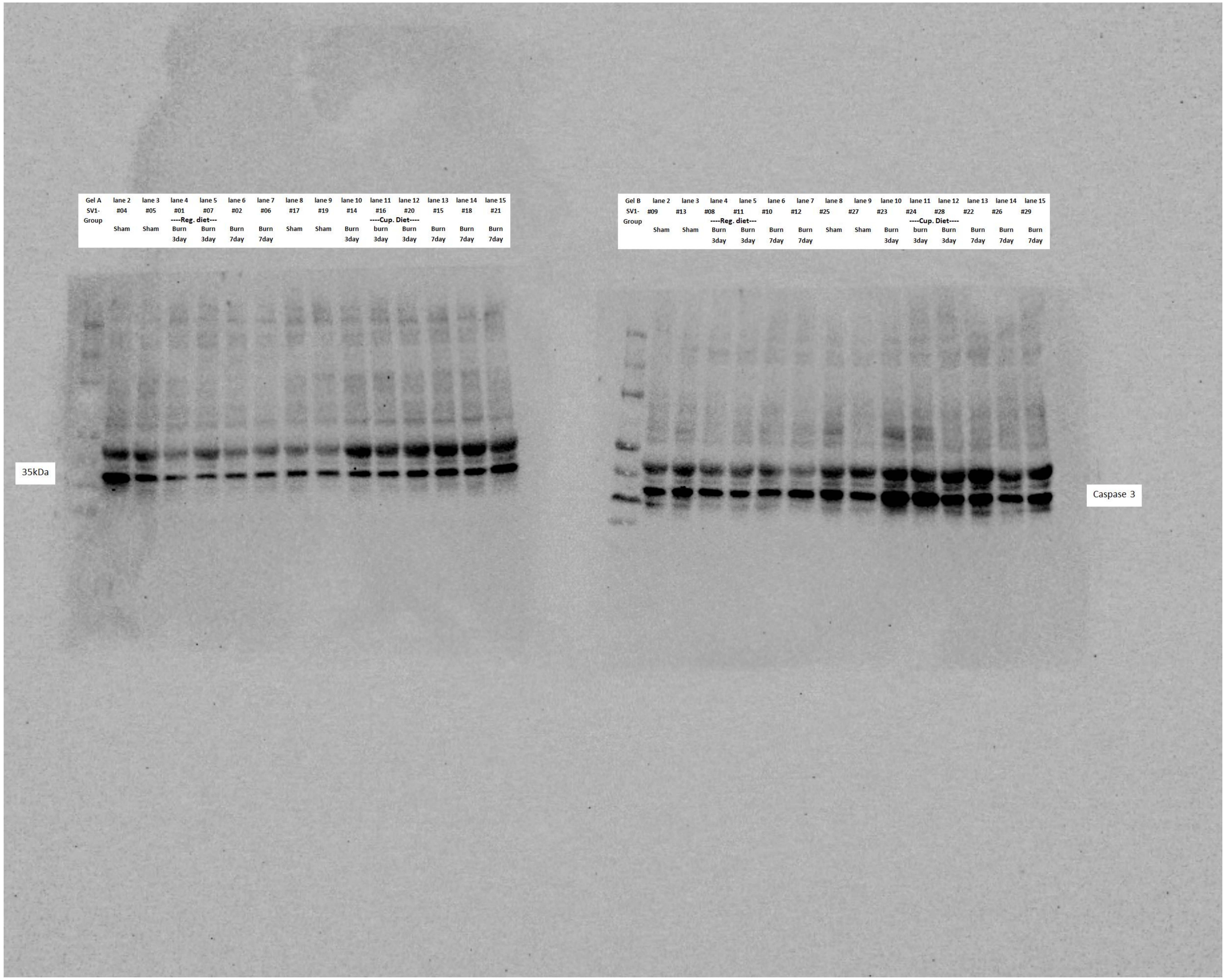

[SongJ 2020-02-26 11h47m07s-sv1-gstr-caspase3]

Fig 4A

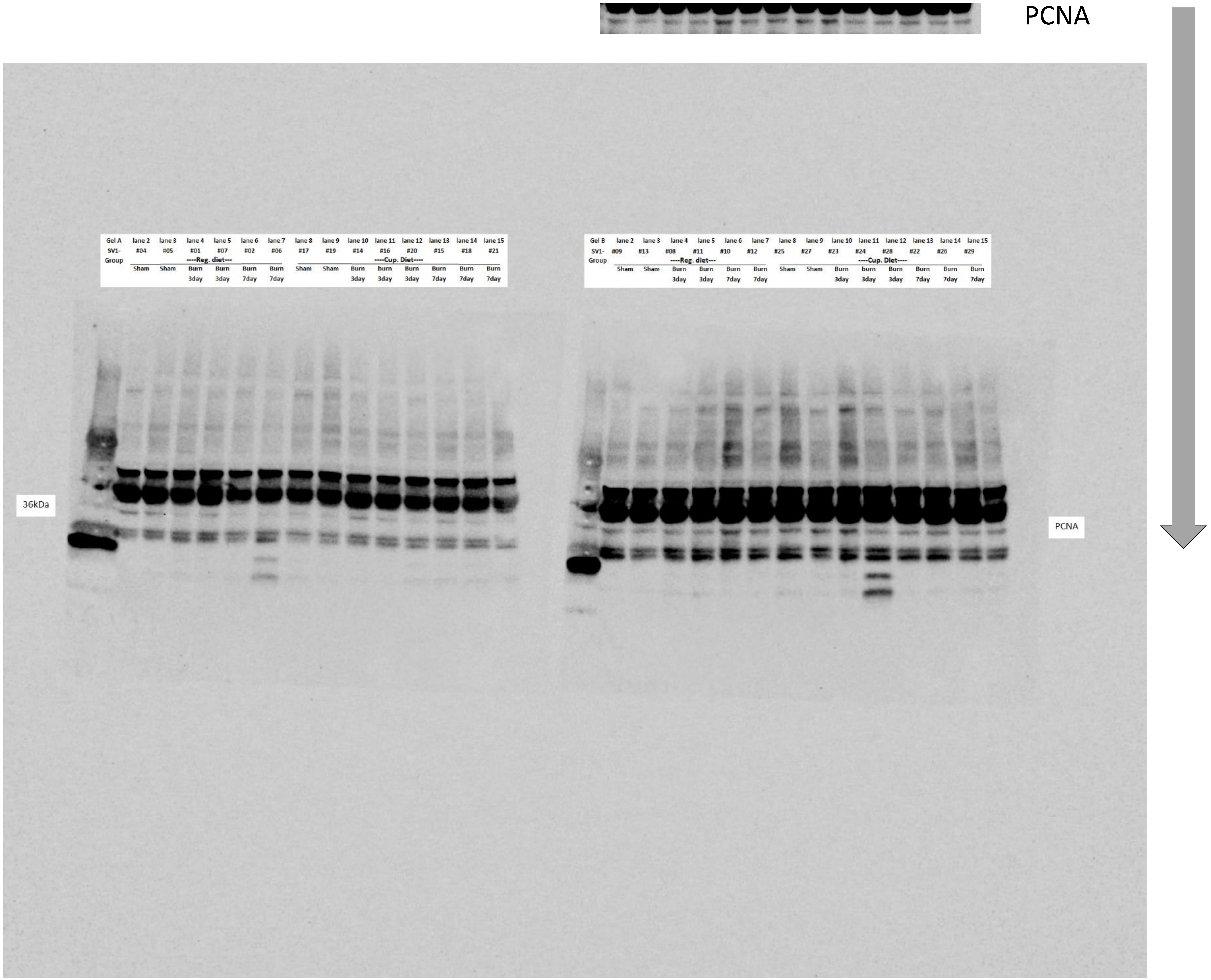

[SongJ 2020-02-28 11h35m46s-sv1-gastr-pcna]

Fig 4A

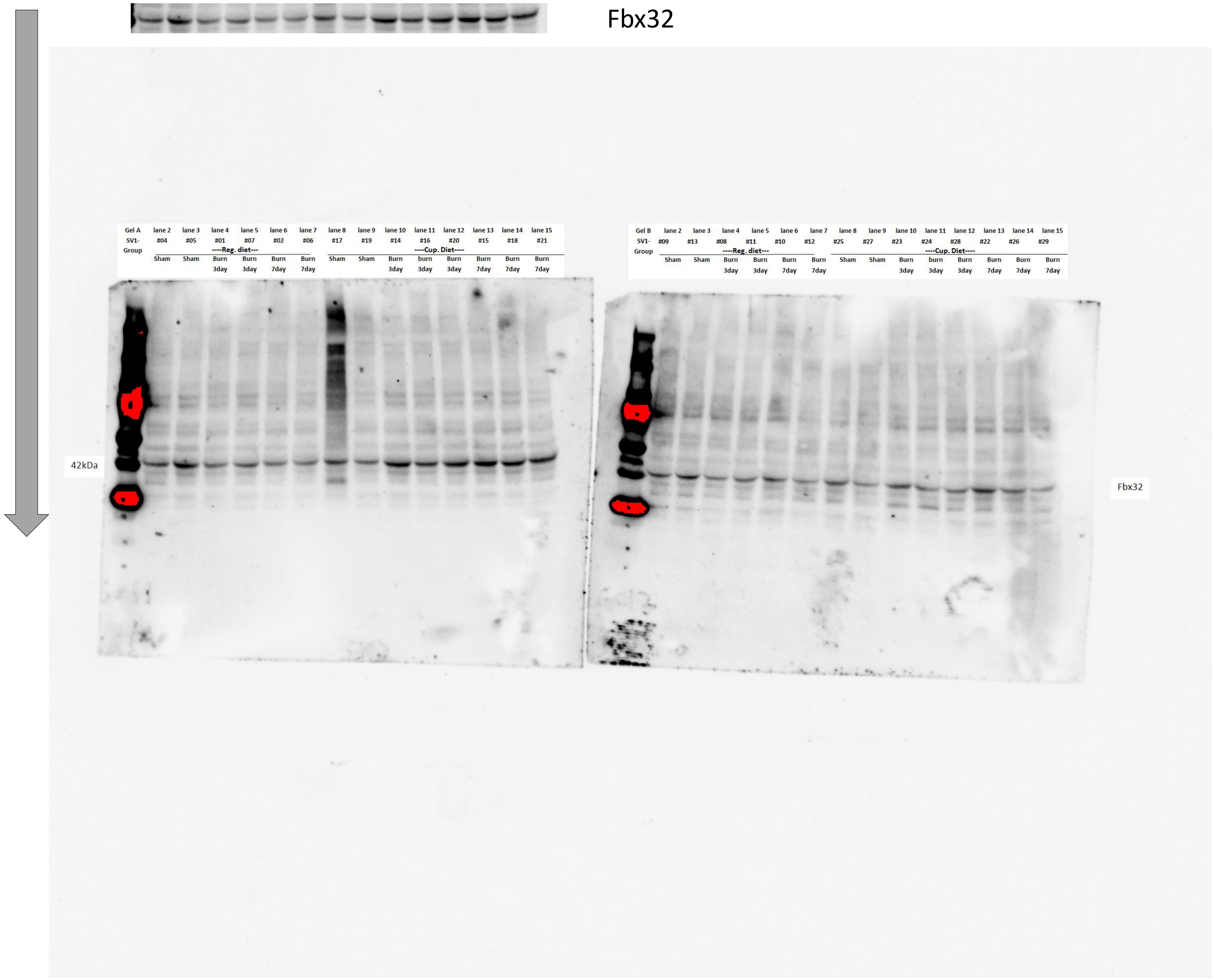

[SongJ 2020-03-02 11h56m29s-sv1-fbx32]

Fig 4A

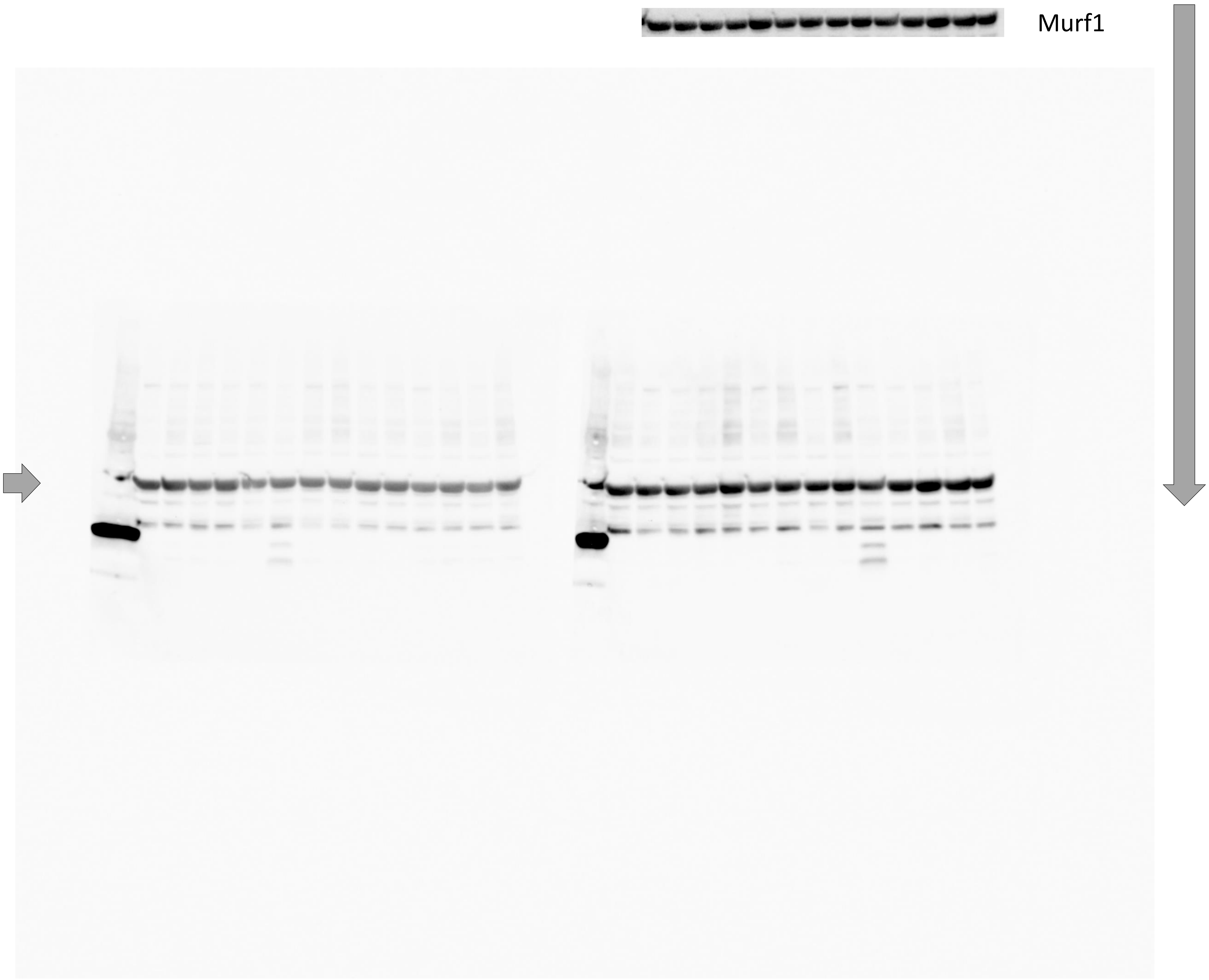

[SongJ 2020-02-27 10h38m24s-sv1-gastr-murf1]

Fig 4A

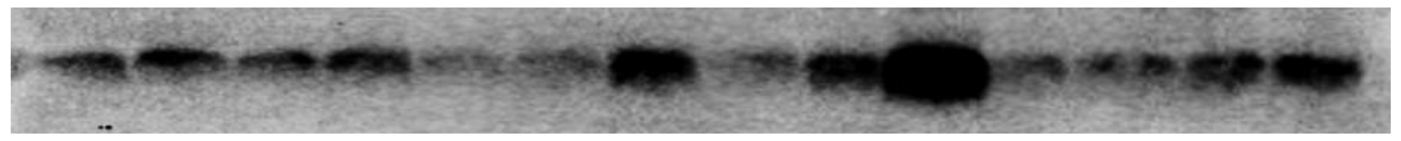

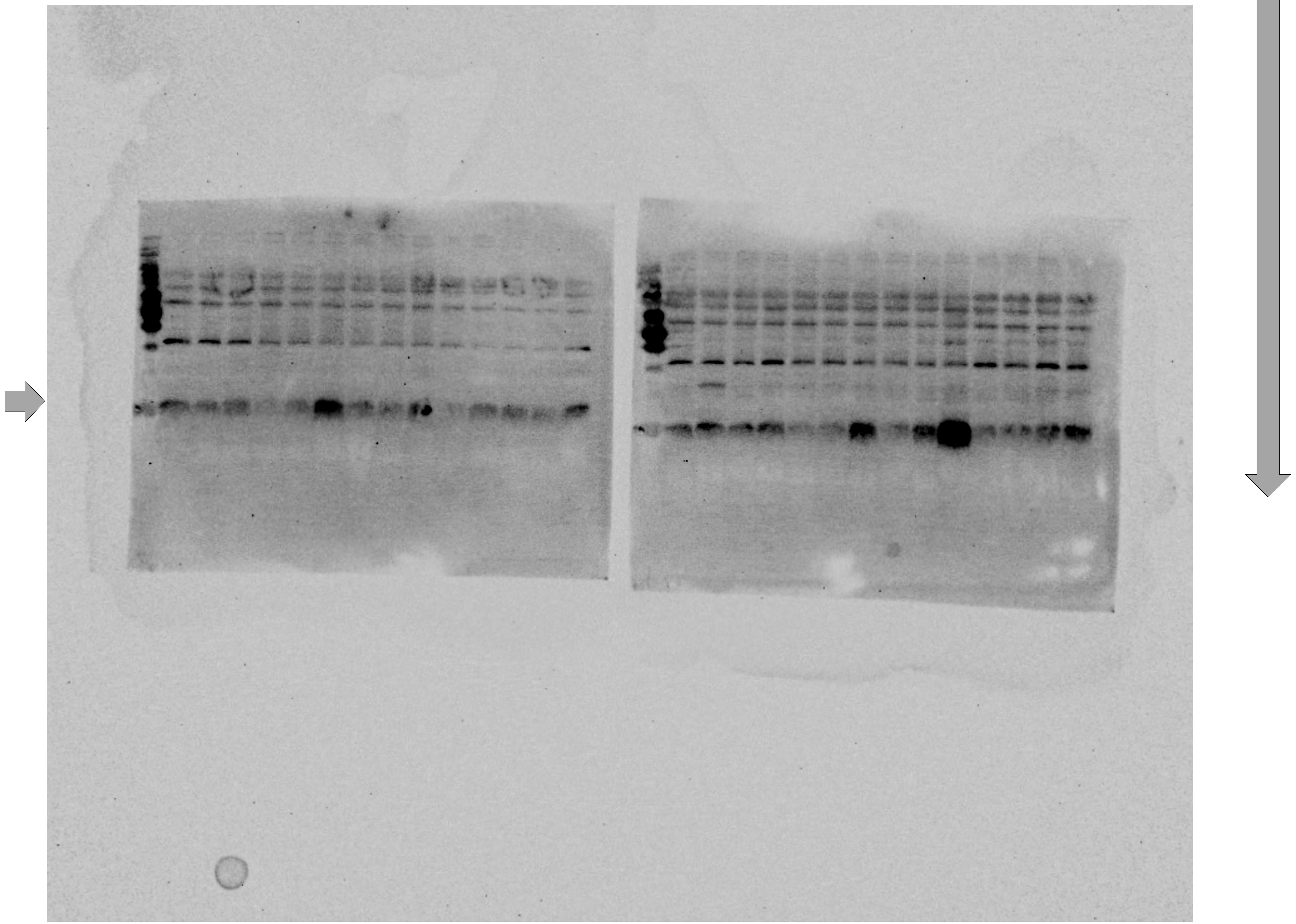

[SongJ 2020-03-06 11h41m15s-sv1-18%-S100b]

Fig 4A

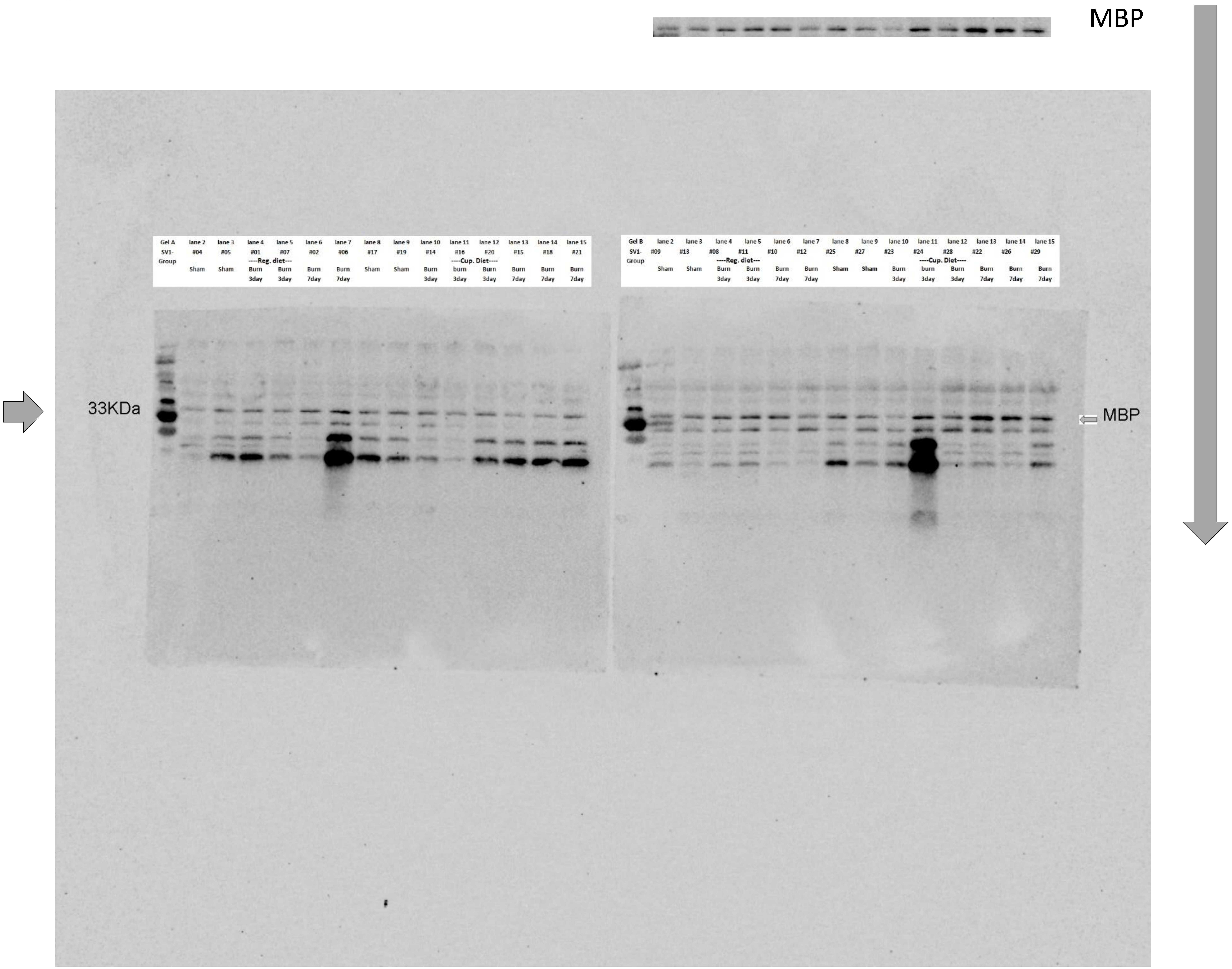

[SongJ 2020-03-09 11h10m11s-sv1-18%-mbp]

Fig 4A

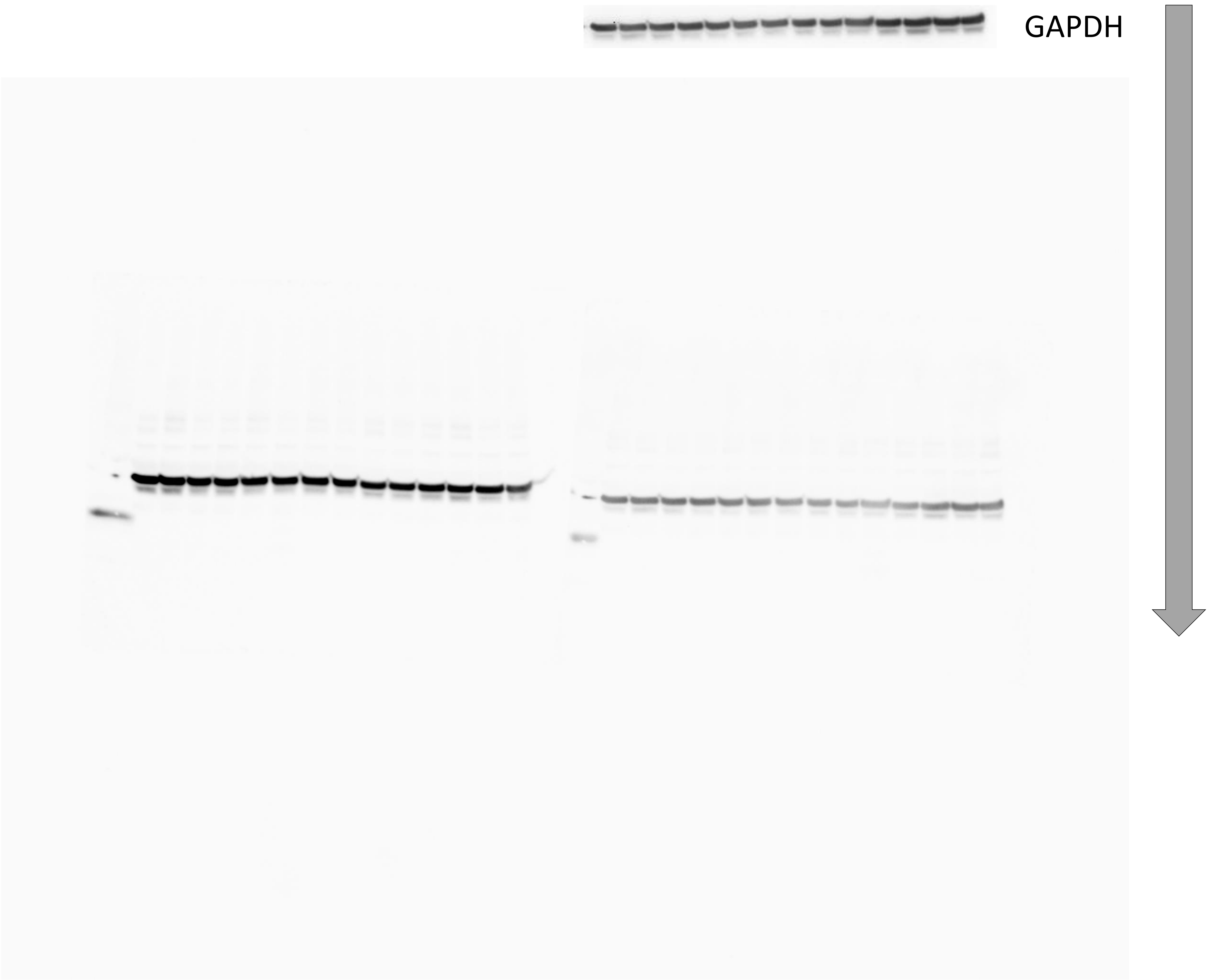

[SongJ 2020-03-02 11h49m41s-sv1-gapdh]
